# Supplementary figures and images for: Paternal adverse childhood experiences and offspring’s attentional disengagement from faces at 8 months—Results from the FinnBrain Birth Cohort Study
Source: PLoS One. 2025 Jul 3;20(7):e0326437. doi: 10.1371/journal.pone.0326437 (PMC12225828; doi:10.1371/journal.pone.0326437)

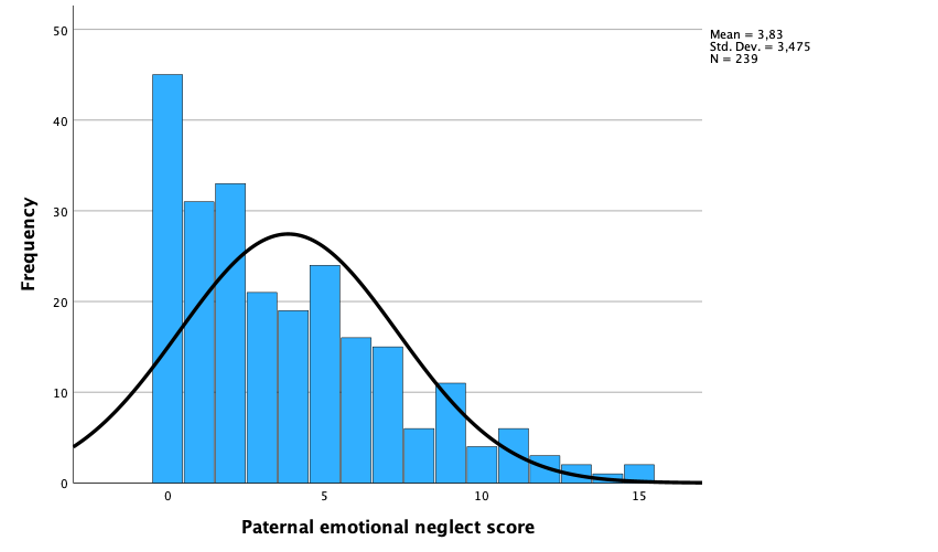

Supplement: S1 File — S1 Fig. The distribution of paternal emotional neglect score. S2 Fig. The distribution of paternal emotional abuse score. S3 Fig. The distribution of paternal physical neglect score. S4 Fig. The distribution of paternal physical abuse score. S5 Fig. The distribution of paternal sexual abuse score. S6 Fig. Relationship between the paternal total TADS score and the offspring’s face bias with paternal anxiety level at 6 months postpartum as a mediator. S7 Fig. Relationship between the paternal sexual abuse score and the offspring’s face bias with paternal anxiety level at 6 months postpartum as a mediator. (ZIP) [file pone.0326437.s001.zip › ESM_PACE Corrected/Fig.S1.tif]

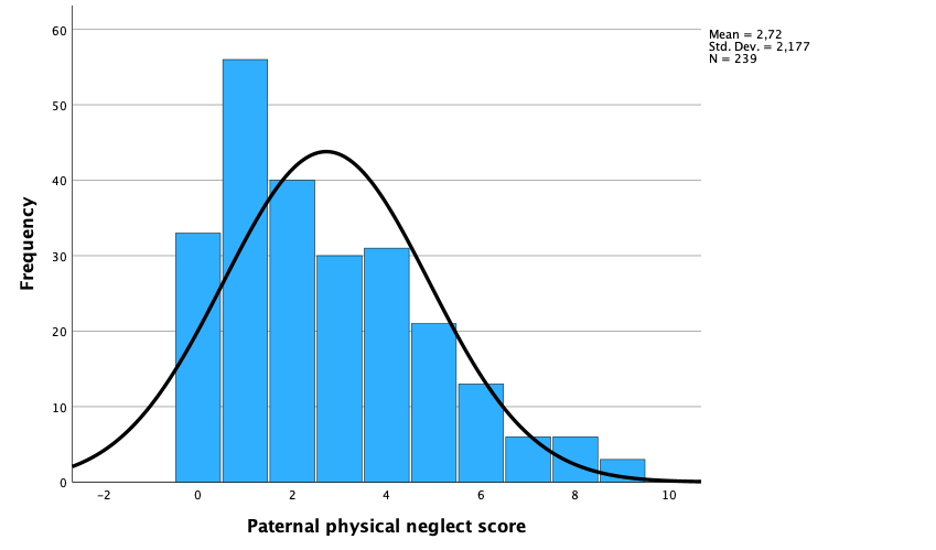

Supplement: S1 File — S1 Fig. The distribution of paternal emotional neglect score. S2 Fig. The distribution of paternal emotional abuse score. S3 Fig. The distribution of paternal physical neglect score. S4 Fig. The distribution of paternal physical abuse score. S5 Fig. The distribution of paternal sexual abuse score. S6 Fig. Relationship between the paternal total TADS score and the offspring’s face bias with paternal anxiety level at 6 months postpartum as a mediator. S7 Fig. Relationship between the paternal sexual abuse score and the offspring’s face bias with paternal anxiety level at 6 months postpartum as a mediator. (ZIP) [file pone.0326437.s001.zip › ESM_PACE Corrected/Fig.S3.tif]

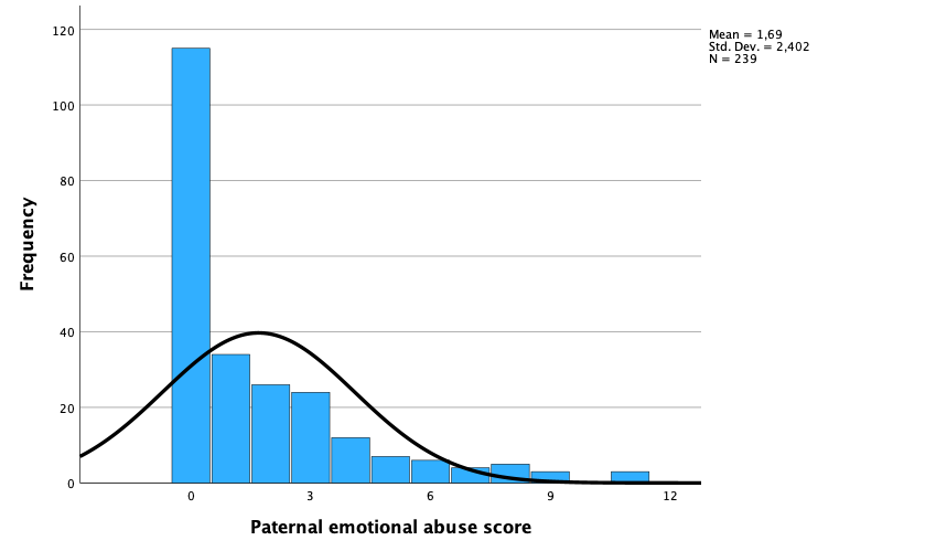

Supplement: S1 File — S1 Fig. The distribution of paternal emotional neglect score. S2 Fig. The distribution of paternal emotional abuse score. S3 Fig. The distribution of paternal physical neglect score. S4 Fig. The distribution of paternal physical abuse score. S5 Fig. The distribution of paternal sexual abuse score. S6 Fig. Relationship between the paternal total TADS score and the offspring’s face bias with paternal anxiety level at 6 months postpartum as a mediator. S7 Fig. Relationship between the paternal sexual abuse score and the offspring’s face bias with paternal anxiety level at 6 months postpartum as a mediator. (ZIP) [file pone.0326437.s001.zip › ESM_PACE Corrected/Fig.S2.tif]

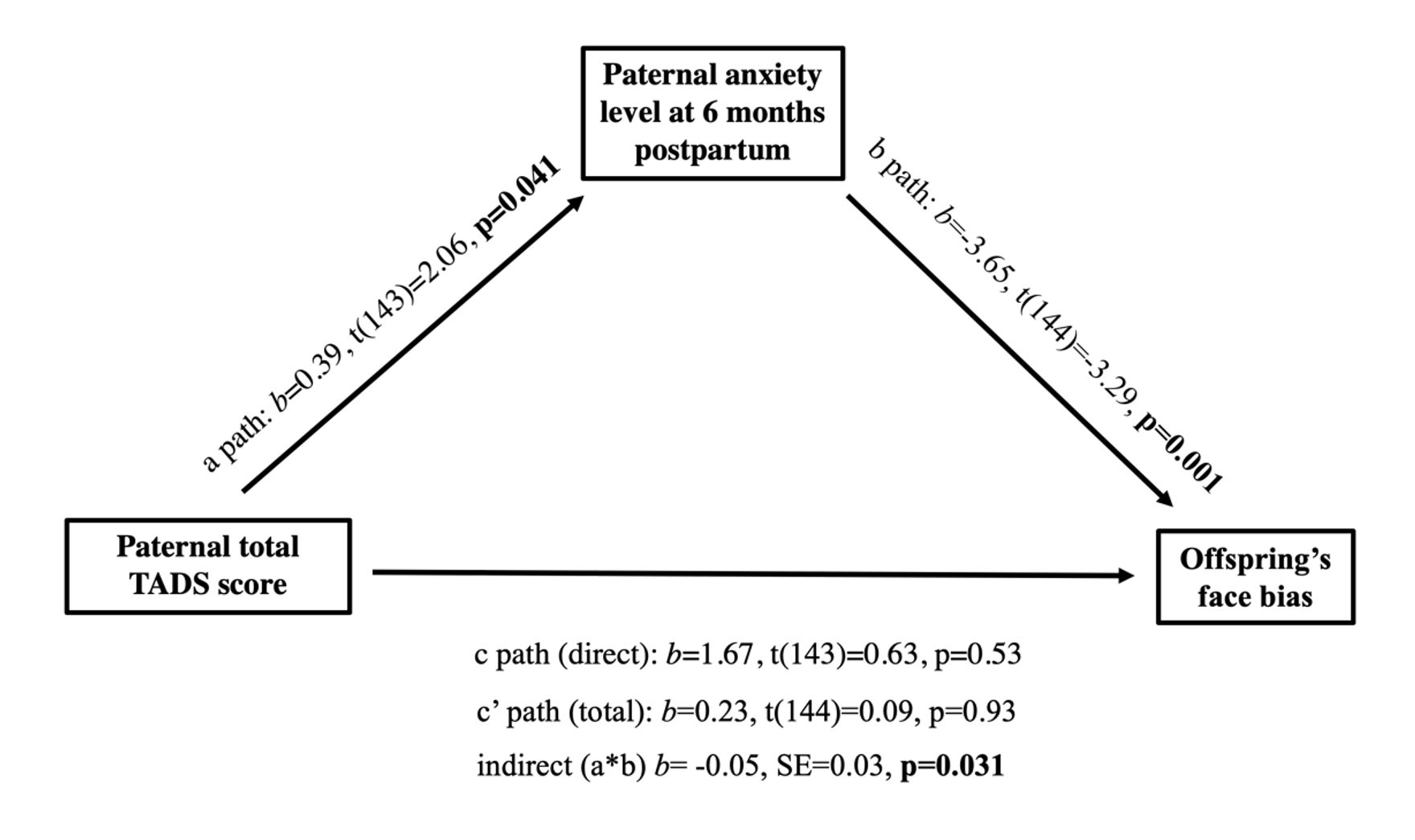

Supplement: S1 File — S1 Fig. The distribution of paternal emotional neglect score. S2 Fig. The distribution of paternal emotional abuse score. S3 Fig. The distribution of paternal physical neglect score. S4 Fig. The distribution of paternal physical abuse score. S5 Fig. The distribution of paternal sexual abuse score. S6 Fig. Relationship between the paternal total TADS score and the offspring’s face bias with paternal anxiety level at 6 months postpartum as a mediator. S7 Fig. Relationship between the paternal sexual abuse score and the offspring’s face bias with paternal anxiety level at 6 months postpartum as a mediator. (ZIP) [file pone.0326437.s001.zip › ESM_PACE Corrected/Fig.S6.tif]

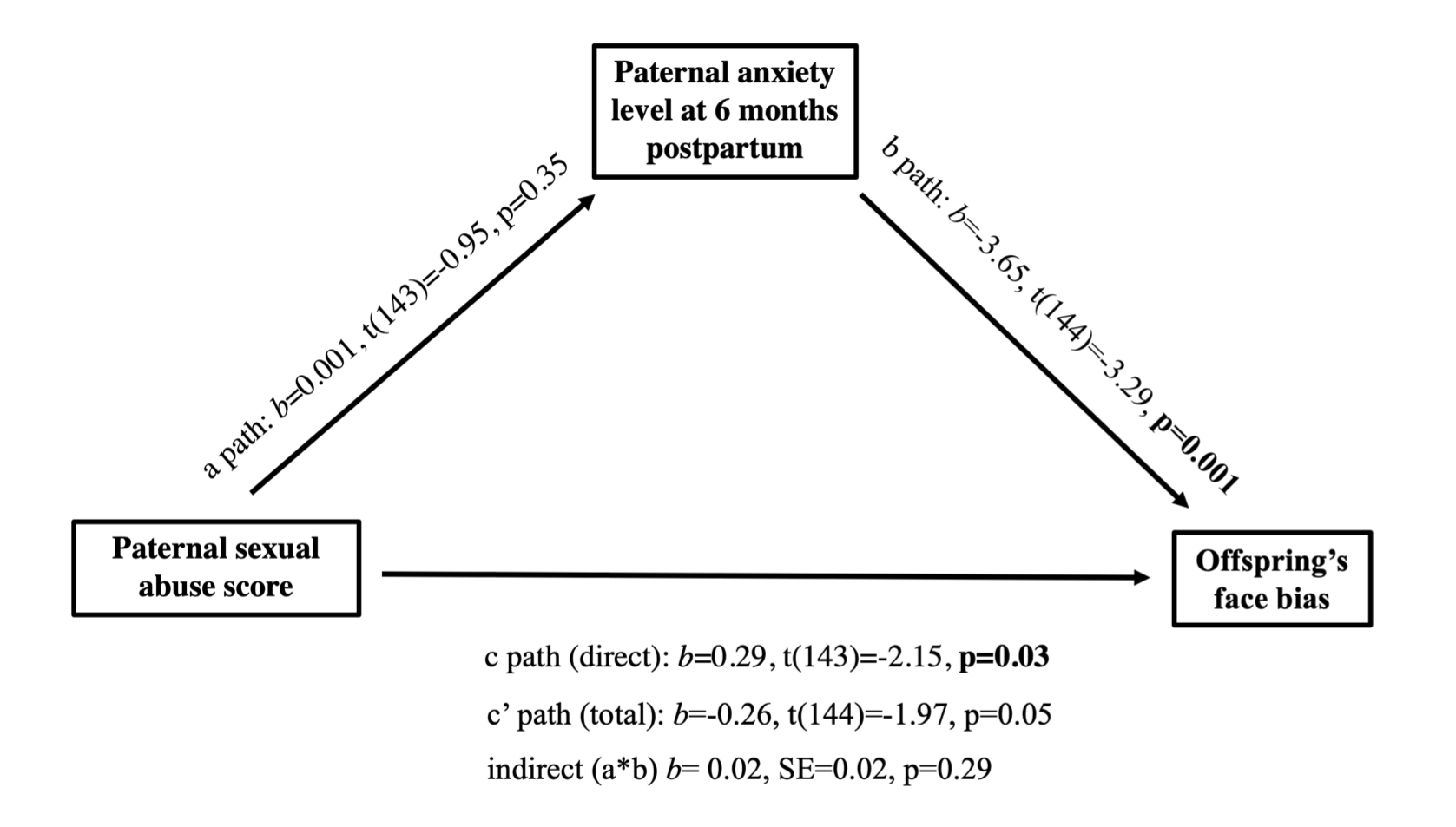

Supplement: S1 File — S1 Fig. The distribution of paternal emotional neglect score. S2 Fig. The distribution of paternal emotional abuse score. S3 Fig. The distribution of paternal physical neglect score. S4 Fig. The distribution of paternal physical abuse score. S5 Fig. The distribution of paternal sexual abuse score. S6 Fig. Relationship between the paternal total TADS score and the offspring’s face bias with paternal anxiety level at 6 months postpartum as a mediator. S7 Fig. Relationship between the paternal sexual abuse score and the offspring’s face bias with paternal anxiety level at 6 months postpartum as a mediator. (ZIP) [file pone.0326437.s001.zip › ESM_PACE Corrected/Fig.S7.tif]

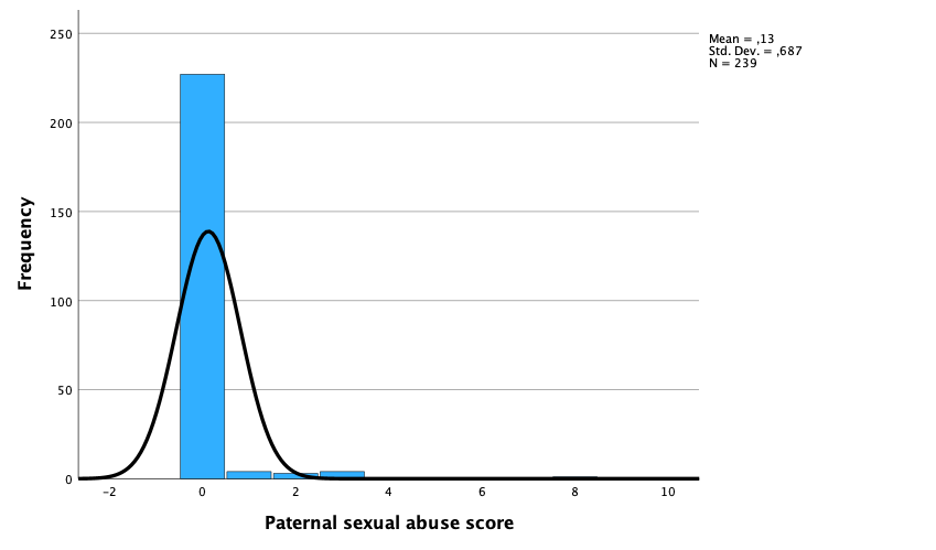

Supplement: S1 File — S1 Fig. The distribution of paternal emotional neglect score. S2 Fig. The distribution of paternal emotional abuse score. S3 Fig. The distribution of paternal physical neglect score. S4 Fig. The distribution of paternal physical abuse score. S5 Fig. The distribution of paternal sexual abuse score. S6 Fig. Relationship between the paternal total TADS score and the offspring’s face bias with paternal anxiety level at 6 months postpartum as a mediator. S7 Fig. Relationship between the paternal sexual abuse score and the offspring’s face bias with paternal anxiety level at 6 months postpartum as a mediator. (ZIP) [file pone.0326437.s001.zip › ESM_PACE Corrected/Fig.S5.tif]

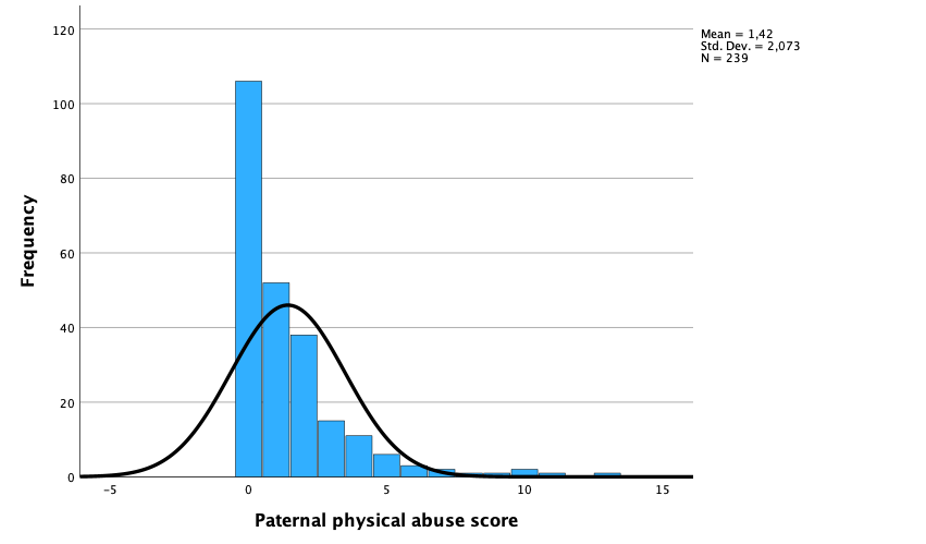

Supplement: S1 File — S1 Fig. The distribution of paternal emotional neglect score. S2 Fig. The distribution of paternal emotional abuse score. S3 Fig. The distribution of paternal physical neglect score. S4 Fig. The distribution of paternal physical abuse score. S5 Fig. The distribution of paternal sexual abuse score. S6 Fig. Relationship between the paternal total TADS score and the offspring’s face bias with paternal anxiety level at 6 months postpartum as a mediator. S7 Fig. Relationship between the paternal sexual abuse score and the offspring’s face bias with paternal anxiety level at 6 months postpartum as a mediator. (ZIP) [file pone.0326437.s001.zip › ESM_PACE Corrected/Fig.S4.tif]
