# Supplementary material for: Paternal adverse childhood experiences and offspring’s attentional disengagement from faces at 8 months—Results from the FinnBrain Birth Cohort Study
Source: PLoS One. 2025 Jul 3;20(7):e0326437. doi: 10.1371/journal.pone.0326437 (PMC12225828; doi:10.1371/journal.pone.0326437)
Supplement: Attachment — Submitted filename: Response_to_reviewers.docx [file pone.0326437.s002.docx]

**Response to Reviewers**

*Paternal adverse childhood experiences and offspring’s attentional disengagement from faces at 8 months – results from the FinnBrain Birth Cohort Study*

**PONE-D-24-53637**

*We sincerely thank the Reviewers for their valuable input, which has greatly improved the clarity and rigor of our manuscript. We have carefully considered each suggestion and revised the manuscript accordingly, whenever it was possible. We trust that these revisions address the Reviewers' concerns. Below, we provide a point-by-point response to all comments. All changes made to the manuscript are highlighted using tracked changes.*

**Reviewer #1:** The study by Klimek and colleagues is interesting and touches a very vital topic Paternal ACE on intergenerational effects on attention at very early stage of neurodevelopment. While Brain carries the signature of ACE from early childhood because ACE has the maximum effect (Please see Kashyap et al, Psychological Medicine) on the brain (compared to all other factors), but whether the effects are carried out to next generation is definitely interesting and required. The methodology is also simple and easily understandable to readers. I have few issues that can be handled easily.

***Response:*** *Thank you for pointing our attention to the interesting paper by Kashyap et al. (2024) on the effects of ACEs on brain developmental patterns. The analysis of neurodevelopmental trajectories of fathers exposed to ACEs and potential transgenerational effects of such exposure is a very desired avenue for future studies in this area.*

2.I believe the introduction can be shortened- Keeping the readers focus to the work done and shortening the Genetic-neuroimaging parts

***Response:*** *The Introduction has been shortened as suggested.*

3.Is there anyway to check for similarity of faces between the presented stimulus and faces encountered easily- (possibly done by any questionaires to parents)- I understand that the manuscript is from a concluded experiment, but still (else may be a limitation)

***Response:*** *Unfortunately, we did not collect any additional data on how easily faces were encountered by children; therefore, we cannot compare the similarities as suggested. Such information was added to the “Limitations of the study”.*

4.Please define each model - (in equation form) above its table. Also provide more parametric values from the tests for model validity.

***Response:*** *Each model was defined in a form of equation as suggested. Additional values (t-statistics and standard error) of the tests performed were added to Table 3.*

5.I would like to see a more focussed discussion pertaining to the outputs of the study- Most of the studies they have cited can go in future direction including genetics , neuroimaging etc.

***Response:*** *In the Discussion, we aimed to do our best to review the plausible reasons for the observed results, also in light of our previous studies. As our study is exploratory by nature, we were only able to speculate about the potential embeddings of the observed associations and the potential mechanistic pathways. For now, we cannot discuss the study's outputs in more detail, as further research in this area is highly needed. We strongly hope the Discussion is informative and potentially proposes valuable avenues for future research.*

Minor-
Spelling mistakes exist
Shorten the introduction and discussion

***Response:*** *The manuscript was again checked for spelling and grammar errors. We have also shortened the text, especially in the Introduction section.*

**Reviewer #2:** Thank you for inviting me to review this manuscript. The authors attempt to relate paternal ACEs to offspring’s attentional disengagement from faces at 8 months. Below are my comments for the authors:

• How were the 1,953 fathers from the FinnBrain Birth Cohort Study selected? For example, was this a random or convenience sample? The same question applies to the 239 children. Including a flowchart would be helpful.

***Response:*** *This sample is a Cohort sample; all eligible participants were contacted in the area of the city of Turku and Åland Islands (Southwest Finland). This information was added to the manuscript. We also added the Flowchart (now Figure 1) as suggested.*

• Table 1 is somewhat limited. Do you have any additional information about your sample? For example: whether fathers are still in a relationship, whether children were born prematurely, any relevant medical history of the children, parents’ education levels, parents’ psychiatric history, childcare arrangements, family history, etc.

***Response:*** *We have now added information about premature birth and parental education level as requested. Regarding the childcare arrangements, the children included in this study were 8 months old. In Finland, only older children (up from 9 months of age) are eligible to attend daycare facilities, therefore we do not have such information. We have also checked the status of the relationship of the participants (n=239) and found that only one father reported separation at 6 months after the child was born. Moreover, the prevalence of mental health problems is very low in this sample. Only 3 fathers exceeded the clinical threshold for anxiety, 10 fathers reported depression and none of them had psychosis based on self-report assessment at the time of recruitment to the study (12 gwk) and at 6 months post-partum. It is crucial to underscore that those conditions were self-reported. Moreover, we do not collect data on diagnoses verified by a medical doctor within our data collection. Therefore, given the low numbers of affected fathers (a possible underreporting) and self-reporting nature of the data, we did not include this information in the manuscript. We now mention the fact of potential underreporting of mental health problems of the fathers in the “Limitations of the study” section.*

• On page 13, you state: “There was no difference between the studied group and the total sample, etc.” Does this comparison refer to the 239 fathers versus the 1,953 fathers? What about comparisons with all fathers from the FinnBrain Birth Cohort Study?

***Response:*** *Yes, indeed, this comparison refers to the 239 fathers (data on eye-tracking available) vs the 1953 fathers. The n=1953 is the number of fathers recruited with the FinnBrain Birth Cohort Study for whom data on adverse childhood experiences were known (collected via the TADS questionnaire). The total number of fathers recruited within the FinnBrain study is N=2623. We have now added the information about the total number of fathers to the manuscript (and also to the requested flowchart). Currently, to explore the representativeness of the study sample (n=239), we compare the paternal age at birth, estimated paternal income, paternal depression and anxiety level at 6 months postpartum between the n=239 fathers and the total number of fathers recruited within the study (N=2623) as requested. There was no difference between the studied group (n=239) and the total sample (N=2623) in paternal age at birth (p=0.56), estimated paternal income (p=0.31), paternal depression (p=0.69), and anxiety level (p=0.37) at 6 months postpartum.*

• On page 14: “If a statistically significant correlation was observed, then the covariate was included in the final statistical model.” This approach is not ideal. Covariate selection should be based on a conceptual framework, independent of statistical correlation.

***Response:*** *Thank you for drawing our attention to this fact. We explored the selected covariates closely and detected several significant correlations between them, many of them of a nonnegligible magnitude (e.g. between maternal and paternal age at birth r=0.694; p<0.001; between paternal anxiety and depression at 6 months post-partum r=0.655; p<0.001; between maternal anxiety and depression at 6 months post-partum r=0.593; p<0.001; between paternal anxiety and maternal depression at 6 months post-partum r=0.196; p=0.02 or between maternal age at birth and family income r=0.167, p=0.01). Therefore, to avoid collinearity, prevent overfitting, and to avoid the potential decrease in statistical power, we would prefer to keep the models as simple as possible.*

• Depression and anxiety at 6 months postpartum may be on the causal path between paternal ACE and attention to faces in offspring. Including these mediators as covariates in your model could bias your causal estimate.

***Response:*** *We do agree that paternal postnatal mental well-being can surely act as a potential mediator between paternal ACEs and offspring’s attentional disengagement from faces. In our manuscript (Discussion section), we present a mediation analysis as well, including the paternal anxiety level at 6 months post-partum as a mediator. The analysis is described as follows:*

*(Lines 492-502): “We performed a mediation analysis to explore if paternal postnatal anxiety levels could act as a mediator of the observed association between paternal ACEs and offspring’s face bias as well as between paternal sexual abuse during childhood and offspring face bias. This analysis was exploratory by nature and was not originally hypothesized. We indeed revealed an indirect association between paternal TADS score (independent variable) and offspring’s face bias (dependent variable) mediated by paternal anxiety level at 6 months postpartum (p=0.031 for indirect pathway) (Figure S6, Electronic Supplementary Materials). However, we did not observe a significant indirect association between paternal sexual abuse during childhood and offspring’s face bias mediated through paternal anxiety level at 6 months postpartum (p=0.29 for indirect pathway) (Figure S7, Electronic Supplementary Materials).”*

*Furthermore, for the purposes of this review, we have performed additional analysis including paternal depression at 6 months post-partum as a potential mediator. Yet no statistically significant associations were observed between paternal TADS and offspring face bias indirectly via paternal depression at 6 months post-partum as a potential mediator (p=0.46). Similarly, we have also not observed indirect associations between paternal TADS and offspring face bias via maternal depression at 6 months post-partum as potential mediator (p=0.90).*

*When we analyzed paternal sexual abuse, no indirect association was observed between paternal sexual abuse and offspring face bias mediated through paternal depression at 6 months post-partum (p=0.95). Lack of such association was also observed when maternal depression at 6 months post-partum was employed as mediator (p=0.82).*

*Summarizing, as neither of the parental postnatal mental health parameters act as mediator when analyzing offspring face bias, we would prefer to maintain those parameters as covariates in the models.*

• Along these lines, your Table 3 is subject to the so-called “Table 2 fallacy” (https://pubmed.ncbi.nlm.nih.gov/23371353/), especially regarding paternal anxiety and maternal EPDS being on the causal path.

***Response:*** *As we report above, in our analyses we include parental mental well-being parameters both as covariates and mediators. We present an indirect association between paternal TADS score and offspring’s face bias mediated by paternal anxiety level at 6 months postpartum (p=0.031 for the indirect pathway) in the Electronic Supplementary Materials. Maternal EPDS did not act as a mediator in our analyses (results reported in this response letter). We aimed to do our best in performing and interpreting our results from different points of view without misinterpretation.*

• Still related to your causal framework, your model may not adequately account for confounders. For example, paternal ACEs may be influenced by factors that also affect children’s attentional disengagement, such as family history of addiction or mental illness. Ideally, you would conduct additional analyses to account for these factors. If these data were not collected, this limitation should be acknowledged.

***Response:*** *We do agree that family environment can be a crucial factor related to both paternal ACEs and children’s attentional disengagement. We include the paternal mental well-being parameters (anxiety, depression) in our analyses, as covariates and also as mediators. We have also checked our data in terms of paternal substance addiction – only 1 father reported alcohol addiction, and none of the fathers reported drug addiction. Given the fact that paternal mental health disorders (including substance addiction) had a very low prevalence in the studied group (potentially due to underreporting), we cannot perform the reliable analyses as requested. We acknowledge this fact, and the limitation pointed out by the reviewer is now mentioned in the “Limitations of the study” section.*
